# 17α-Ethinylestradiol (EE2): concentrations in the environment and methods for wastewater treatment – an update

**DOI:** 10.1039/d2ra00915c

**Published:** 2022-04-27

**Authors:** Marko Klaic, Franz Jirsa

**Affiliations:** Department of Inorganic Chemistry, University of Vienna Waehringer Str. 42 1090 Vienna Austria franz.jirsa@univie.ac.at; Department of Zoology, University of Johannesburg Auckland Park 2006 Johannesburg South Africa

## Abstract

17α-Ethinylestradiol (EE2) is a frequently used drug and an endocrine disruptive substance. Adverse effects on biota have been reported when they are exposed to this substance in the environment. The last review on EE2 in the environment was published in 2014. Since then, well above 70 studies on EE2 and related substances have been published. The aim of this review was therefore to bring together recent data with earlier ones. The topics emphasized were observable trends of environmental levels of EE2 and methods to reduce EE2 levels in wastewater, before it can enter the environment. This should give an overview of the recent knowledge and developments regarding these environmental aspects of EE2. In the studies discussed, EE2 levels in surface waters were well detectable in many countries, both above and below the predicted no effect concentration (PNEC) of 0.035 ng L^−1^, although analytical methods used for the quantification often are unsatisfactory regarding their limit of detection. To support the degradation of EE2 prior to entry into the environment, appropriate treatment methods could help to control the emissions of EE2. Several methods for the reduction of EE2 levels of up to 100% removal efficiency were reported recently and are of chemical, biological, adsorptive or ion-exchange nature. Depending on the required properties like initial EE2 concentration or treatment duration, several promising methods are available.

## Abstract 2

17α-Ethinylestradiol (EE2) is a frequently used drug and an endocrine disruptive substance.

## Graphical abstract

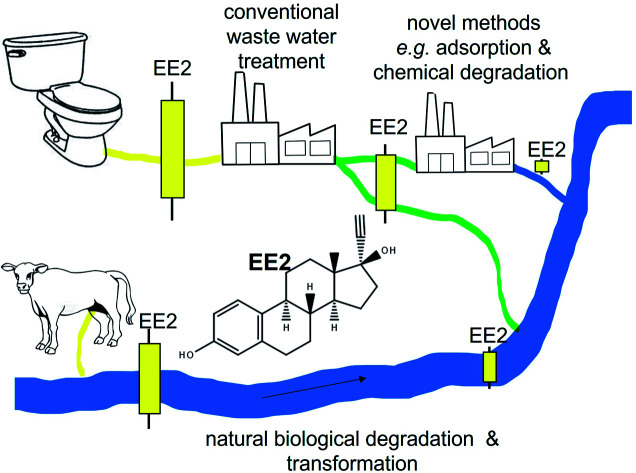

## Introduction

Medicinal products for human and veterinary use are applied in large quantities worldwide. Approximately 4000 active pharmaceutical ingredients, the substances which are supposedly accountable for the desired effect, were available in 2014.^1^ And there are more to come: The European Medicines Agency (EMA) released 66 positive opinions on human medicines and 15 on veterinary medicines in 2019, of which in total 35 were new active substances for that year.^2,3^

After the application of medicinal products their respective pharmacokinetics lead to the release of metabolites or even original substances into the environment. Thus, pharmaceuticals are introduced into surface and groundwater, originating from (treated) wastewater, manure or sludge. The question whether these substances are likely to have an impact on biota in the aquatic environment still is unanswered for many of them.^4^ In the best cases, waste waters are treated in waste water treatment plants (WWTPs) which are designed to remove all, or the major part of pollutants from waste water. The overall operating principle of WWTPs consists of mechanical separation, biological treatment using microorganisms, chemical treatment and polishing. The efficacy of the biological treatment in WWTPs is highly dependent on functioning microbial communities and disturbances may occur, when waste water contains antimicrobial or other substances that might hinder the functionality of these communities. A number of studies showed that medicinal products or their metabolites may as well have a negative impact on the efficacy of WWTPs. Even more, if not negatively affected, WWTPs often are not able to remove these chemicals or their metabolites to a desired degree.^4^ One of the substance groups, which have caused concern in the environment, are hormonally active substances, better known as Endocrine Disrupting Chemicals (EDC). The World Health Organization (WHO) published an assessment titled “State of the Science of Endocrine Disrupting Chemicals” in 2013 to discuss effects of these substances on organisms. EDCs in general are substances, which interact with the endocrine system and cause negative effects on health.^5^ According to the assessment, in 2012 approximately 800 substances were suspected or known for their potential to act as EDCs. Simultaneously, illnesses related to disturbances in the endocrine system have been reported to be increasing: Almost 40% of young men had reduced semen quality, male babies faced increasing numbers of genital malformations, numbers of babies with reduced birth weight and pretermature births were increasing, as well as increasing cases of type 2 diabetes and obesity were observed. It is known that EDCs can affect other biota than humans negatively resulting in numerous ways, *e.g.* causing population declines, increase in endocrine-related disorders *etc.*^6^ One of the substances, which is, without a doubt, an EDC, is 17α-ethinylestradiol (EE2).

**Fig. 1:**
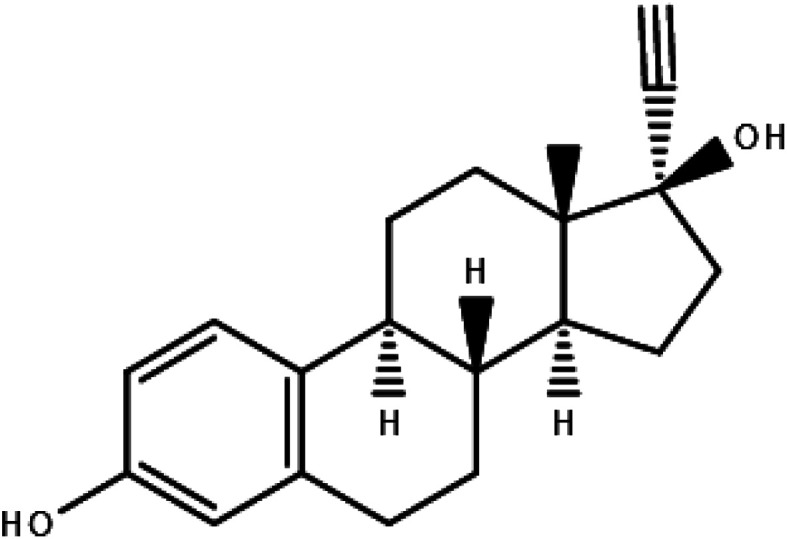
Structure of EE2.

The chemical structure of EE2 is depicted in Fig. 1; the substance was described for the first time in 1938 by Inhoffen and Hohlweg,^7^ who were conducting studies on the efficacy of orally administered, supposedly estrogenically active substances. Studies on a castrated rat, a female baboon and a rabbit delivered positive results on the oral absorption of estrogenic substances, which was a big deal at that time, since hormonally active substances had to be administered *via* injections and it was known already that patient compliance can correlate with more pleasant applications. It took several more years until the development of orally applied contraceptives started in 1950 and it took more than ten more years, until in the 1960s sufficiently functioning oral contraceptives were available, also commonly known as “the pill”. Nowadays usually EE2 is combined with a second substance with a progestogenic effect, leading to products known as “combined oral contraceptive” to enhance the contraceptive effect of EE2. The first pills contained doses of up to 150 μg EE2 but this was reduced to 30 μg per pill in the 1970s.^8^ Products with an even lower content of *e.g.* 20 μg EE2 per pill are also available. During the course of time other beneficial effects of products containing EE2 were observed, *e.g.* using a combined product with drospirenone resulted in reduced menstruation pain or reduced forming of acne.^6^

**Fig. 2:**
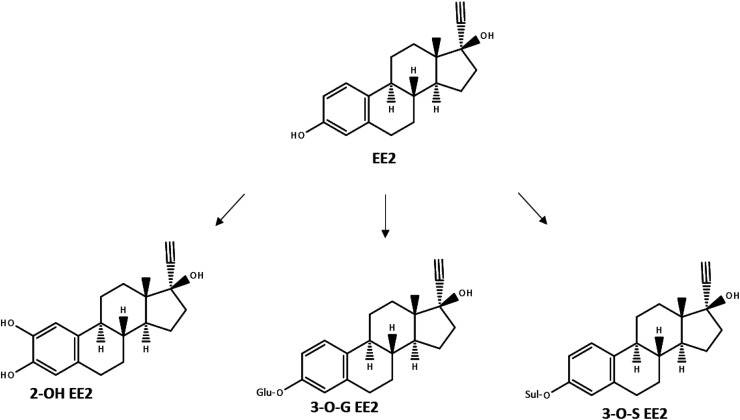
Metabolic pathways of EE2, redrawn after Zhang *et al.* (2007).^9^

As many active ingredients in pharmaceuticals, EE2 is metabolized in the human body after uptake, before it is excreted. It has been reported that major human metabolic pathways of EE2 are hydroxylation *via* CYP enzymes, glucuronidation or sulfation.^9^ In all three cases it is visible that the core structure is still existing and that the chemical group which was added onto the core structure of EE2 could be separated again (Fig. 2).

The wide-spread use of drugs containing EE2 as affective ingredient was followed by a massive release of EE2 and its metabolites into the environment. Aris *et al.* (2014) named the main sources of EE2 in the environment: Human urine, livestock wastewater and runoffs of manure and sewage sludge which was used previously agriculturally.^10^

Once in the environment adverse effects have been observed in biota. A drastic example was given by Hoffmann and Kloas (2012) who showed the harming potential of EE2 on the frog species *Xenopus laevis*.^11^ Adult males which were exposed to various concentrations of EE2 displayed lowered sexual arousal, which was clearly shown by analyzing their calls. This result was an obvious indication of how EE2 can have an impact on biota in the environment, which could lead, in the worst case, to extinction of a population because of mating loss. In the review article published by Aris *et al.* (2014) topics like EE2 levels found in the environment, effects of EE2 on exposed organisms and the possible removal of EE2 were discussed.^10^ Detectable environmental levels of EE2 and different effects on various species were reported. The need to propose a specific design to eliminate EE2 in the environment was expressed. Although research groups around the world have since then addressed this topic, some of these many questions remain open. In this work we try to give an overview of the current situation of research and address the most urging questions regarding levels of EE2 in the environment and novel solutions to successfully remove EE2 from wastewaters.

### Levels of EE2 in the environment

The EU is currently monitoring several substances in their member states which are considered as emerging pollutants.^12^ In April 2018, a document containing the 1^st^ review of this “Watch List by the European Commission's science and knowledge service”, the Joint Research Centre was published. 25 member states have submitted data for this compilation, while Spain, Greece and Malta did not submit any data at all. Of the submitted data, 98.3% were river samples, 1.2% lake samples and 0.5% coastal/transitional water samples.^13^ One of the substances which was monitored was EE2.

Without looking at the measured concentrations yet, one parameter catches the attention of the reader: The desired Limit of Quantitation (LOQ) is set equally to the predicted no effect concentration (PNEC), below which no effects are expected. For EE2 the PNEC is estimated to be 0.035 ng L^−1^ (ref. 12) a concentration which many laboratories delivering data for the Watch List review could not achieve as their LOQ. Four countries reported that the commissioned laboratories reached an LOQ of 0.03 ng L^−1^, another 4 countries reported 0.035 ng L^−1^, all other countries reported values above these levels. 82 samples were therefore to be quantified and of those, in 75 the PNEC of 0.035 ng L^−1^ was exceeded. Nonetheless, the authors tried to utilize the data with higher LOQs than 0.035 ng L^−1^ and developed two scenarios to interpret all available data: In both scenarios, values for samples which could not be quantified were set to half of the respective LOQ. For further prediction, two cases were developed: In one case, all records were considered, in the other case only records were considered if the recalculated value was equal or below the PNEC. Only in the latter case, the median concentration in environmental samples was 0.015 ng L^−1^ and thus below the PNEC. The other calculations led to median concentrations of 0.05 ng L^−1^ and 0.1 ng L^−1^, respectively, both exceeding the PNEC. Eventually the authors concluded that the available data is insufficient and therefore the substance should remain on the watch list.^13^ Interestingly, EE2 is not found on the updated watch list, which was installed in 2020.^14^

An overview of results of EE2 levels in environmental samples, published in recent years is given in Table 1. One of the EU member states which has provided data is Austria, and this member state has also published its own report regarding the presence of hormones and pharmaceuticals in surface waters. The report states that 20 measuring points were selected in Austria and from each, a sample was taken in fall/winter 2017 and another sample in spring 2018. The samples were analyzed using a method which detected multiple substances at the same time using ultra high-performance liquid chromatography connected with a tandem mass spectrometer (UPLC-MS/MS). The outcome was, that EE2 was not detected in any sample, but at the same time the method showed a LOQ of 0.1 ng L^−1^ and a limit of detection (LOD) of 0.05 ng L^−1^. Both limits are well above the PNEC of 0.035 ng L^−1^. Additionally, the estrogenic effect of the samples was determined using a bioassay and the results of this assay lead to the assumption that even if EE2 was not detectable analytically, it was probably present at low concentrations in the samples.^15^ In January 2010, the team around Valdés *et al.* took samples from sewage effluents and their respective receiving waters in the Pampas region and the Río de la Plata estuary in Argentina to determine the concentration of estrone, 17β-estradiol and EE2.^16^ In total seven samples were analyzed using HPLC-MS/MS with a LOD of 15 ng L^−1^ and LOQ of 45 ng L^−1^ for EE2. EE2 was detected in every sample of sewage effluent accounting to 80 ng L^−1^, 65 ng L^−1^ and 187 ng L^−1^ respectively. In the surface waters only one sample had detectable levels of EE2 (43 ng L^−1^) - the concentration in the three other samples was below the LOD. The authors interpreted the results as a probable threat to aquatic organisms.^16^ The determination of the distributions of estrogens and bisphenol A in the Yangzte River Estuary in China and the East China Sea was the aim of Shi *et al.*, who took water and sediment samples in the wet season in 2010 and in the dry season in 2011. Four municipal WWTPs discharge their effluents into the Yangtze River Estuary; this was considered in the sampling strategy by distribution of the sampling points. Samples were analyzed *via* LC-MS/MS and the method detection limits were ranging from 0.02 to 0.05 ng g^−1^ for sediment samples and from 0.02 to 0.1 ng L^−1^ for water samples. In the wet season, EE2 levels were not detectable in all 15 water samples and 30 sediment samples. In the dry season, EE2 was detected in only one out of 30 water samples (0.11 ng L^−1^) and in two out of 30 sediment samples (0.06 ng g^−1^ and 0.72 ng g^−1^). In a separate recalculation to determine the estrogenicity of a drawn sample where the sampling location is close to a livestock farm, the high estrogenic potential was assigned to high EE2 levels in the sample.^17^ Nie *et al.* took samples in August 2011 from the upper Huangpu River, a large river in Shanghai to analyze EE2 as well as the other estrogenic compounds estrone, estradiol, estriol, bisphenol A and 4-*tert*-octyphenol. While five sampling sites were located at five tributaries of the Huangpu River, six sampling sites were located at the main river itself and additional four sampling sites were taken from receiving streams of animal feeding operations. Suspended particulate matter and colloidal samples, obtained by filtration, were analyzed as well to identify if there was a tendency for adsorption. The chosen method for the analysis of EE2 was GC-MS using a prior derivatization step. The authors report LODs of 0.10–0.49 ng L^−1^ and LOQs of 0.30–1.97 ng L^−1^ in aqueous samples and LODs of 0.15–0.44 ng g^−1^ and 0.93–3.15 ng g^−1^ in suspended particulate matter samples for all analyzed substances. In the aqueous samples, EE2 was detected at concentrations up to 20.1 ng L^−1^ from animal feeding operation receiving streams, while except for one single sample of the main river, EE2 was not detectable. This was also the case for colloidal samples, EE2 was detected in only one sample. On the contrary, EE2 was detected in all suspended particulate matter samples with levels of up to approximately 120 ng g^−1^. In this case the highest values were observed in the samples taken at the receiving stream of animal feeding operations.^18^ The northern parts of the Taihu Lake in China were the study area of Wang *et al.* in May 2013 in which various estrogenous compounds were investigated, one of those was EE2. Eight sampling sites were chosen and water, sediment and biota (fish, river snail and clam) samples were collected and analyzed using HPLC-MS/MS. The LODs were 0.8 ng L^−1^ for water samples and 0.5 ng g^−1^ for sediment samples as well as for fish samples, a specific LOD for other biota samples was not stated, assuming same limits due to the identical sample preparation. In water samples, EE2 was only detectable in two samples with 21.1 ng L^−1^ and 33.5 ng L^−1^. EE2 was detectable in every sediment and biota sample and ranged from 4.32 to 184 ng g^−1^ in sediment samples and from 21.3 to 417 ng g^−1^ (dry weight) in biota. In direct comparison of the three biota species, river snails displayed a bioaccumulation factor (BCF) of 25 033 for EE2, for clams it was 6061 and for fish 4115, respectively.^19^ The authors demonstrated the tendency of EE2 to accumulate in sediment with a concentration of up to 184 ng g^−1^ and levels up to 417 ng g^−1^ in biota. Reasons for this behavior have been described from other lipophilic substances. Looking into the metabolism of EE2 this can be explained easily: EE2 itself is a highly lipophilic substance which is excreted mainly *via* conjugation with hydrophilic groups (Fig. 2); which guarantees higher solubility in urine. If those groups are separated from the mother substance during physico-chemical or biological degradation, the highly lipophilic behavior returns. This leads to a high affinity to *e.g.* organic substances in sediment or fat containing compartments in biota.^19^ Prior to the previously described study, the team around Yan *et al.* took samples from the same waterbody, Taihu Lake in China.^20^ In contrast to Wang *et al.* the samples were taken earlier in the year, from November to December in 2011. The focus in this study were in general emerging organic contaminants, among those was also EE2. They used a combined UHPLC/MS/MS and achieved an LOD of 1 ng L^−1^ for EE2. At two out of eight sampling points, EE2 was not detectable. The concentration range of the other substances was between 1.64 ng L^−1^ and 4.00 ng L^−1^. An additional calculation of the hazard quotient lead to the result that EE2 was one of the greatest hazards in the lake.^20^ The results obtained from Wang *et al.* and Yan *et al.* are the results which can be compared the best since the waterbody was the same and many sampling locations were close to each other. The sampling took place approximately one and half years apart and therefore it is possible to compare the course. One result draws the attention of the reader: The concentration at the inlet to the Wangyu river in 2011 was at 2.28 ng L^−1^ and in 2013 at 21.1 ng L^−1^, respectively. This means, that the concentration had risen approximately nine-fold in only 1.5 years, assuming these results are not outliers. The highest reported value in 2011 was 4.00 ng L^−1^, and the highest reported value in 2013 reached 33.5 ng L^−1^. Although a trend regarding rising EE2 concentrations is visible, it must be kept in mind that singular points in time are not able to portray the whole picture, since various regular and irregular, known and unknown impacts can influence the EE2 concentration. One known impact has been reported for instance by Coelho *et al.*, who described a negative correlation of EE2 concentrations and flow rates, which usually decline naturally during dry periods.^21^

**Table 1:** EE2 levels in the environment (n.d. = not detected), LODs are stated, where given

Sampling location or waterbody	EE2 concentration	Reference
20 rivers in Austria	<LOD (0.05 ng L^−1^)	Loos *et al.*^13^
Huangpu River receiving streams (water), China	n.d. – 20.1 ng L^−1^	Nie *et al.*^18^
Huangpu River receiving streams (colloidal matter), China	App. 5–120 ng g^−1^ dw	
Surface Waters, Pampa Region, Argentina	43–187 ng L^−1^	Valdés *et al.*^16^
Surface Water, Río de la Plata estuary, Argentina	<LOD (15 ng L^−1^)	
Taihu Lake (water samples), China	n.d. – 33.5 ng L^−1^	Wang *et al.*^19^
Taihu Lake (sediment samples), China	4.32–184 ng g^−1^ dw	
Taihu Lake (biota samples), China	21.3–417 ng g^−1^ dw	
Taihu Lake, China	n.d. – 4.00 ng L^−1^	Yan *et al.*^20^
Danube, Budapest, Hungary	0.124 ng L^−1^	Avar *et al.*^24^
Danube, Dunaföldvár, Hungary	<LOD (0.001 ng L^−1^)	
Danube, Solt, Hungary	<LOD (0.001 ng L^−1^)	
Danube, Paks, Hungary	<LOD (0.001 ng L^−1^)	
Danube, Mohács, Hungary	0.005 ng L^−1^	
Drava, Maribor, Slovenia	0.006 ng L^−1^	
Drava, Drávaszabolcs, Hungary	<LOD (0.001 ng L^−1^)	
Sava, Ljubljana, Slovenia	0.002 ng L^−1^	
Ljubjanica, Ljubljana, Slovenia	0.003 ng L^−1^	
Mur, Murarátka, Hungary	0.008 ng L^−1^	
Zala, Balatonhídvég, Hungary	0.68 ng L^−1^	
Hévíz-Páhoki canal, Alsópáhok, Hungary	0.52 ng L^−1^	
Imremajori canal, Balatonfenyves, Hungary	0.018 ng L^−1^	
Sió, Szekszárd-Palánk, Hungary	0.097 ng L^−1^	
Kapos, Kaposvár, Hungary	<LOD (0.001 ng L^−1^)	
Zagyva, Szolnok, Hungary	<LOD (0.001 ng L^−1^)	
Tisza, Szolnok, Hungary	<LOD (0.001 ng L^−1^)	
Tisza, Tiszakécske, Hungary	0.099 ng L^−1^	
Tisza, Csongrád, Hungary	0.143 ng L^−1^	
Lake Balaton, Balatonlelle, Hunary	0.133 ng L^−1^	
Lake Balaton, Balatonszárszó, Hungary	<LOD (0.001 ng L^−1^)	
Lake Balaton, Tihany, Hungary	<LOD (0.001 ng L^−1^)	
Pécsi víz total, Pécs, Hungary	0.175 ng L^−1^	
Guadiamar River, Spain	<MDL (15.0 ng L^−1^)	Garrido *et al.*^25^
Hawkesbury River, Australia	n.d. – 29 ng L^−1^	Uraipong *et al.*^27^
Huai River, China	n.d. – 0.174 ng L^−1^	Niu and Zhang^23^
8 rivers in Portugal	<LOD (6.82 ng L^−1^)	Pereira *et al.*^28^
Laguna de Rocha, Uruguay	<LOQ 0.1 μg L^−1^	Griffero *et al.*^29^
Laguna de Castillos, Uruguay	n.d. – 45 μg L^−1^	
Billings Reservoir Branch, Brazil	n.d. – 1200 ng L^−1^	Coelho *et al.*^21^
Yangtze River Estuary (water samples), China	n.d. – 0.11 ng L^−1^	Shi *et al.*^17^
Yangtze River Estuary (sediment samples), China	n.d. – 0.72 ng g^−1^ dw	
Shenandoah River Watershed, USA	n.d. – 2.4 ng L^−1^	Barber *et al.*^26^

Wang *et al.* and Shi *et al.* analyzed sediment samples in Taihu Lake (China) and the Yangtze River Estuary (both located in China) and detected up to 184 ng g^−1^ dry weight and 0.72 ng g^−1^ dry weight, respectively. As reported by Aris *et al.*, several earlier studies had measured EE2 levels in sediments. The highest reported concentration therein was *ca.* 130 ng g^−1^ dry weight,^22^ while levels in other several sediment samples from China did not exceed 10 ng g^−1^ dry weight. Another China-based study was conducted by Niu and Zhang regarding the Huai River and its potential pollutants. In January 2010 water samples were taken at four different sites. The samples were analyzed using an HPLC system with a diode array detector and reached a LOD of 0.12 pg L^−1^ for EE2. With this comparably low LOD, EE2 was detectable in 11 out of 12 samples in total and the concentration was ranging from 0.048 ng L^−1^ to 0.174 ng L^−1^. The authors pointed out that EE2 had the tendency to decrease in its concentrations along the run of the river.^23^ Comparing older studies with more recent ones from China, the country with the most results reported, it can be observed that rising EE2 concentrations were detected over time. Avar *et al.* published a study, in which samples from rivers in the Carpathian Basin in Slovenia and Hungary were measured to determine concentrations of estradiol and EE2. Unfortunately, it was not stated when the samples were taken. The authors used HPLC-MS and HPLC-MS/MS as the analyzing system, the LOQ for EE2 was 0.001 ng L^−1^ for HPLC-MS at and 0.2 ng L^−1^ for HPLC-MS/MS, respectively. Both systems were used to determine the EE2 concentration and regarding the HPLC-MS method, several samples had concentrations ranging from 0.002 ng L^−1^ to 0.175 ng L^−1^. The presence of EE2 using HPLC-MS/MS was only confirmed at two sampling sites out of 23, namely at river Zala at Balatonhidvég (0.62 ng L^−1^) and the canal Héviz-Páhoki at Alsópáhok (0.436 ng L^−1^).^24^ Another study which was conducted in an EU member state was published by Garrido *et al.*, who monitored emerging pollutants in the Guadiamar River basin in southern Spain. Among several other substances, EE2 was analyzed in water samples which were collected in June 2014 at six sampling sites, which were located at the main river and tributaries. The analysis was performed using a LC-MS/MS system with a method specific for the determination of hormones and industrial pollutants. For EE2 they achieved a LOD of 15.0 ng L^−1^ and a LOQ of 49.5 ng L^−1^. In the studied area, EE2 was not detected in any sample.^25^ Barber *et al.* conducted a large-scale study with various aims to assess topics like fish endocrine disruption, exposure risk and wastewater reuse in the Shenandoah River Watershed in eastern West Virginia and northern Virginia. Another target was to verify if the model which was used to predict environmental concentrations was close to reality and therefore whether the predicted environmental concentrations equaled measured environmental concentrations. For this verification, samples were taken in 2014, 2015 and 2016 over a period of 4 weeks every 7 days, while the number of samples which was drawn varied between 4 and 12 at each site. The samples were analyzed using 21 different analytical methods, one of those measured among other substances was EE2. The method detection limit was at 0.1 ng L^−1^ and the authors reported the following results: EE2 was measured above the method detection limit at only one out of nine locations at the South Fork Shenandoah River (2.4 ng L^−1^), the concentrations in all other samples were below the LOD.^26^ The Hawkesbury River in Australia was the point of interest in a study published by Uraipong *et al.* The authors developed an ELISA for the specific simultaneous detection of EE2 and mestranol, which was used in a second step to determine the concentration of those substances in water supply. Eight samples were taken along the Hawkesbury River in Emigrant Greek, Northern New South Wales and South Greek, Sydney upstream, at the discharge point and downstream of the discharge point of WWTPs stating that the samples were “fresh” but no sampling period was stated. The researchers were able to achieve a LOD of 0.04 ± 0.02 μg L^−1^ and an LOQ of 0.05 ± 0.01 ng L^−1^ for EE2 and similar results for mestranol. With their method were the following results obtained: Upstream samples of the WWTPs had concentrations of 15 ng L^−1^, downstream samples had concentrations of 28–29 ng L^−1^ for EE2 and mestranol. In a catchment nearby the WWTP, the combined concentrations were in the catchment at 5.5 ng L^−1^, the farer away upwards the samples were taken the concentrations were lower with 8.3 ng L^−1^ at 3 km, 6.1 ng L^−1^ at 9 km and 4.1 ng L^−1^ at 11 km. According to the scientists, although no separation of the combined concentrations of EE2 and mestranol was conducted, they expected that almost 100% of the detected substances were EE2 residues. Another statement that was made, was the expectation that agriculture and urbanization are the main contributors to the high EE2 levels.^27^ The aim of the study by Pereira *et al.* was to give an insight on the impact of surface water flow rates and WWTPs on environmental concentrations of various substances, among them EE2. The samples were taken across Portugal from Tâmega River, Tua River, Mondego River, Trancão River, Tagus River, Xarrama River, Guadiana River and Álamo Creek. From 20 different sites in 2014 from September to November and in 2015 from February to March, altogether 72 samples were collected. The analysis was performed using a LC-MS/MS system, for EE2 the method detection limit was at 6.82 ng L^−1^ and the method quantification limit at 20.65 ng L^−1^. EE2 was not detected in any analyzed sample.^28^ Griffero *et al.* published a study about South American Atlantic coastal lagoons in Uruguay. At 23 points of the Laguna de Castillos and the Laguna de Rocha samples were taken along streams, lagoons and coastal sea zones. Sampling took place in February, May, August and November 2017 and resulted in a total of 92 samples. The analysis was performed using LC combined with high resolution mass spectrometry, the LOQ for EE2 was 0.1 μg L^−1^. EE2 was detectable only in four samples: In May at one site with 0.24 μg L^−1^, in August at the same site with 0.13 μg L^−1^ and in winter at two different sites with 0.42 μg L^−1^ and 45.51 μg L^−1^. Even though in general temporal distribution for the other substances was not observed, EE2 levels increased in winter at a site close to an urban area according to the authors.^29^ For the study of Coelho *et al.*, the researchers took samples in São Paulo waters in Brazil, namely in one of the Billings reservoir branches. In total eight sampling campaigns took place from June 2017 to February 2018, four in the dry period from June to August 2017 and four in the wet period from October 2018 to February 2019. The analysis was performed according to a method published by the USEPA *via* LC-MS with a limit of detection of 30 μg L^−1^ and limit of quantification of 100 μg L^−1^. The authors state that by evaporation of the solvent and suspension of the residue, a concentration factor of 1000 was achieved leading to a quantification limit of 100 ng L^−1^. In the dry period, EE2 concentrations were ranging from <LOQ to 1200 ± 140 ng L^−1^ while in the wet period the concentrations were ranging from <LOQ to 300 ± 90 ng L^−1^. At one sampling site with low anthropogenic impact no EE2 was detectable, the other three sampling sites were probably impacted by WWTPs, according to the researchers.^21^ The highest EE2 level in water samples was measured in Brazil with 1200 ± 140 ng L^−1^. This exceeds the PNEC by a factor of over 300 000. The authors of this study propose that the dry season in which this sample was taken had an influence on environmental concentrations of EE2.^21^ This effect was also observed by Shi *et al.* from China, although the concentration range in their study was between not detectable and 0.11 ng L^−1^.^17^ Earlier analyses of water samples were also summarized by Aris *et al.*^10^ with a highest reported EE2 level of 34 ng L^−1^ at the Venice Lagoon, Italy.^30^ This value was by far exceeded in the recent study from Brazil, in which 1200 ng L^−1^ were reported.^21^ European levels reported in the last years were comparatively lower, but these results should be taken with precaution, since some European laboratories operated with LODs as high as 6.82 ng L^−1^ or 15.0 ng L^−1^, respectively.^25,28^

### EE2 in biota

Results for levels of EE2 in biota from natural environments are scarce. Recent publications include works by Wang *et al.*^19^ from Taihu Lake and Zhang *et al.*^31^ from Yundang Lagoon in Xiamen City, both in China. Zhang *et al.* report their results based on the lipid weight. EE2 levels were 3.42 ng g^−1^ in the short-necked clam *Ruditapes philippinarum* sample, 3.03 ng g^−1^ in the black seabream *Acanthopagrus schlegel* and 2.71 ng g^−1^ in the yellow fin seabream *Sparus latus*, whereas in samples from a tilapia species EE2 was below the detection limit of 0.54 ng g^−1^.^31^ Wang *et al.* in contrast reported concentrations in biota ranging from 21.3 to 417 μg kg^−1^ dry weight.^19^ For a better comparison, concentrations presented by Zhang *et al.* in lipid weight were recalculated referring to dry weight using data on total lipids and water provided by the United States Department of Agriculture Food Data Central Database (https://fdc.nal.usda.gov/). According to this approximation, the EE2 content in *R. philippinarum* was 0.16 μg kg^−1^, in *A. schlegel* 0.34 μg kg^−1^ and in *S. latus* 0.30 μg kg^−1^, all referring to dry weight, respectively. These recalculated values are far below the values which were reported by Wang *et al.* It has to be kept in mind, that these singular measurements are just snapshots of a moment, nonetheless a direct comparison points to an increase in EE2 concentration over time. Earlier studies from 2007 and 2010, respectively, had reported EE2 levels up to 38 ng g^−1^ dry weight in mussels^30^ and up to 11.34 μg kg^−1^ dry weight (recalculated from wet weight given in the paper) in fish.^32^ These contents of EE2 are well comparable to the results by Wang *et al.*in 2015.^19^

### Methods to reduce EE2 levels in waste water treatment plants

Due to comparable mechanisms, methods can be grouped in “Chemical treatment”, “Biological treatment” and “Adsorption and Ion Exchange”. A list of treatment methods which lead to the best reported removal efficiencies is given in Table 2.

**Table 2:** Methods to reduce EE2 levels

Treatment method which lead to the best reported removal efficiency	Best reported removal efficiency, treatment-period in brackets	Initial EE2 concentration	Ref.
KMnO_4_ and ultrasound	70.5% (120 min)	25 μg L^−1^	Deng *et al.*^33^
UVC, H_2_O_2_ and ultrapure water	100% (10 min)	100 μg L^−1^	Frontistis *et al.*^34^
Nanoscale zero-valent iron	*Ca.* 100% (300 min)	120 μg L^−1^	Jarošová *et al.*^35^
Ultrasonic ozonation	86.0% (12 min)	5 μg L^−1^	Zhou *et al.*^36^
K_3_FeO_4_ or K_2_FeO_4_	100% (5 min)	100 μg L^−1^	Machalová Šišková *et al.*^37^
Natural organic matter and horseradish peroxidase	35.1% (8 h)	500 μg L^−1^	Yang *et al.*^38^
TiO_2_ coated glass rings	*Ca.* 98% (60 min)	20 μg L^−1^	de Liz *et al.*^39^
Modified magnetite, H_2_O_2_	100% (15 min)	1000 μg L^−1^	Serrano *et al.*^40^
Intracellular polymeric substances from anaerobic cultures	75.5% (5 h)	0.5 mg L^−1^	He *et al.*^41^
AgI/BiOI/BiPO_4_	100% (8 min)	3 mg L^−1^	Long *et al.*^42^
Fungal transformation	98.6% (72 h)	10 mg mL^−1^	Różalska *et al.*^43^
*Phoma* sp. strain UHH 5-1-03	*Ca.* 100% (24 h)	74.1 mg L^−1^	Hofmann and Schlosser^44^
Laccase (from *Pycnoporus sanguineus*)	86.18% (4 h)	5 mg L^−1^	Golveia *et al.*^45^
*Shweanella oneidensis*, fulvic acids, sodium anthraquinone-2-sulfonate	41.6% (132 h)	0.5 mg L^−1^	He *et al.*^47^
*Lolium perenne* and *Hyphomicrobium* sp	98.7% (42 d)	23.5 mg kg^−1^	He *et al.*^48^
Long-term electro-domesticated microorganisms, fulvic acids	98.4% (90 min)	0.5 mg L^−1^	He *et al.*^46^
Ryegrass and *Hyphomicrobium* sp. GHH	90% (28 d)	25 mg kg^−1^	He *et al.*^49^
Magnetic ion exchange	75.3% (75 min)	20 μg L^−1^	Wang *et al.*^50^
Sand, vermiculite, charcoal, granulated activated carbon	>99% (30 d)	10 μg L^−1^	de Castro *et al.*^51^
Gamma-cyclodextrin polymer	*Ca.* 100% (5 min)	11.9 μg L^−1^	Tang *et al.*^53^
Soil	*Ca.* 40% (720 min)	2 mg L^−1^	de oliveira *et al.*^54^

### Chemical treatment

The aim of the study conducted by Deng *et al.* was to try a combined oxidation and ultrasound treatment on steroid estrogen mixtures containing E1, E2 and EE2 using potassium permanganate KMnO_4_ as oxidant. The researchers conducted preliminary experiments testing the degradation efficacy of either, only addition of KMnO_4_ or ultrasound treatment. The best reduction efficiency for EE2 (70.5% after 120 min) was observed in 6 mg L^−1^ KMnO_4_ solutions starting at an initial concentration of 25 μg L^−1^ EE2. Further experiments were conducted using lower KMnO_4_ concentration to reduce the colorization of the treated solution, combined with ultrasound treatment. The researchers generated a variety of results, which can be summarized as follows: The removal efficiency increased in combined KMnO_4_ and ultrasound systems, the removal efficiencies were higher in binary estrogen systems compared to the tertiary estrogen system and removal efficiencies were higher in a natural water matrix compared to pure water.^33^ Unfortunately, the researchers decided not to use a higher concentration of KMnO_4_ for additional studies although they reported a positive correlation between removal efficiency and oxidant concentrations. Frontistis *et al.* published a study to investigate the degradation of EE2 by solar radiation, UVA and UVC. In one of the experiments which was performed, the researchers used UVC and varying concentrations of H_2_O_2_ leading to a reduction by 100% from initially 100 μg L^−1^ EE2 using 10 mg L^−1^ H_2_O_2_ and a reaction time of 15 minutes. Using the same initial concentration, various water matrices were tested (ultrapure water, secondary-treated wastewater, 10 mg L^−1^ humic acid solution and a mixture containing same parts of secondary treated wastewater and ultrapure water). For UVC combined with 10 mg L^−1^ H_2_O_2_, complete removal was achieved after 15 min in every matrix, except for ultrapure water were 100% removal efficiency were achieved after 10 min. Interestingly, although removal efficiencies of up to 100% were reported, the authors clearly point out, that estrogenicity might still be present in the samples after treatment, due to the presence of endocrine disruptive degradation products.^34^ The effect of nanoscale zero-valent iron (nZVI) on E2 and EE2 concentrations was the aim of a study published by Jarošová *et al.* They prepared solutions which contained 60 μg L^−1^ E2 and 120 μg L^−1^ EE2 and added nZVI particles. The best result generated was using 6 g L^−1^ nZVI particles, removing 93% of EE2 after 1 h and close to 100% after 5 h. Additional bioassays were used to assess the estrogenic activity and revealed the activity decreased in the first hour of the test, and stagnating thereafter.^35^ In a study published by Zhou *et al.*, experiments on the reduction and removal of different substances including EE2, using ozonation, ultrasonic ozonation and photocatalytic ozonation, respectively, were presented. Experiments were conducted spiking sewage effluents with 5 μg L^−1^ EE2, and treating it with either of the mention methods for 12 minutes. The highest removal efficiency (86.0%) was achieved using ultrasonic ozonation with an addition of 30 μg L^−1^ O_3_ at pH = 9.5. In additional experiments, the presence of humic acids caused a reduction of the removal efficiency.^36^ The capability of Fe^IV^, Fe^V^ and Fe^VI^ to remove different estrogens, one of them EE2, was assessed by Machalová Šišková *et al.* The effluent of a WWTP was used and estrogens were added to reach final concentrations of 100 μg L^−1^, while Na_4_FeO_4_ (Fe^IV^), K_3_FeO_4_ (Fe^V^) and K_2_FeO_4_ (Fe^VI^) were used at 1, 10 and 100 mg L^−1^, respectively. Maximum removal was already achieved after 5 minutes using Fe^V^ and Fe^VI^ species at 10 mg L^−1^. Fe^IV^ displayed the lowest efficiency even when using 100 mg L^−1^ Fe^IV^ only approximately 80% of the EE2 were removed.^37^ The team around Yang at al. investigated the influence of natural organic matter (NOM) and horseradish peroxidase (HRP) on photodegradation of EE2. NOM improved the EE2 removal efficiency compared to solutions containing no NOM. The addition of HRP further increased the efficiency. After 8 h, 35.1% of EE2 were removed in a solution initially containing 500 μg L^−1^ EE2, 5 mgC L^−1^ NOM and 0.01 U mL^−1^ HRP. Only 23.1% of EE2 were removed in a similar solution without HRP.^38^ The aim of the study conducted by de Liz *et al.* was the degradation of E1, E2 and EE2 using so-called glass Raschig rings (hollow cylinders with almost identical diameter and length) coated with TiO_2_. Photolytic and photocatalytic reactions were tested by using a mercury vapor lamp (providing UVA and UVC) and an initial concentration of 20 μg L^−1^ pollutant and a reaction time of 60 min. The addition of the glass rings, which corresponded to app. 200 mg L^−1^ TiO_2_ and the use of UVA resulted in removal efficiencies of up to 98% after 60 minutes. Additional experiments using treated WWTP samples, spiked with EE2, showed that removal was inhibited and reached 50% at an initial concentration of 50 μg L^−1^ after 60 min. The researchers also assessed the degradation products and were not able to detect any after 30 min.^39^ The removal of pharmaceutical pollutants including EE2, using modified magnetite (Fe_3_O_4_-R400) as a catalyst and H_2_O_2_ as oxidant was the aim of a study published by Serrano *et al.* In the experiments, 0.2 g L^−1^ of the catalyst and an amount of H_2_O_2_ which corresponded stoichiometrically to the amount of the substance to be removed at initial concentrations of 1000 μg L^−1^ were added. 100% removal efficiency for EE2 was observed at 50 °C already after 15 min, while complete removal was also observed at lower temperatures but required a longer reaction time. An additional experiment with effluents of WWTPs, spiked with 1000 μg L^−1^ EE2 using an increased catalyst quantity of 2 g L^−1^, resulted in a complete removal of EE2 after 60 to 90 min.^40^ A study which was published by He *et al.* dealt with photosensitive cellular polymeric substances (CPS) to accelerate the photodegradation of EE2. The highest removal efficiency of 75.5% was reported for CPS from anaerobic bacterial cultures at a concentration of 10.0 mgC L^−1^ and an initial concentration of 0.5 mg L^−1^ EE2 using a reaction time of 5 h. Analysis of the degradation products of this reaction showed that the basic structure of EE2 was still intact and either a double bond, a hydroxyl group or a ketone group had been added onto the structure. Further experiments showed that not every fraction of the CPS promoted the degradation of EE2. The contribution of hydroxyl radicals on the degradation success was little, but singlet oxygen ^1^O_2_ was very effective. The ionic strength of the CPS had an impact on the degradation rate and either proteins or amino acids accelerated the degradation.^41^

Long *et al.* prepared a photocatalyst consisting of AgI/BiOI/BiPO_4_ and tested its capability regarding the removal of EE2. Solutions containing 3 mg L^−1^ EE2 were prepared, 5 mg of the catalyst were added and left for 30 min in the dark to reach an adsorption equilibrium, then the reaction was started using a Xe lamp. After 8 min, the removal efficiency using AgI/BiOI/BiPO_4_ reached 100%. Additional experiments showed that the catalyst still had a removal efficiency of 82% after a 5^th^ reuse.^42^

The aforementioned methods used catalysts, chemicals, radiation and soundwaves or combinations of those to achieve the removal of EE2. To evaluate those methods regarding a possible application in future, the investigation and characterization of degradation products, as well as possible byproducts originating from the respective treatment agents must be a priority. If these methods should have a future in an upscaled treatment of EE2 in waste water, the stability of *e.g.* catalysts or the possible detrimental effects of their partial dissolution in the used water system must be carefully considered. The same caution must be taken if a follow-up treatment of the byproducts might be necessary. Also, sufficient supply of the required chemicals should be guaranteed to ensure a seamless treatment process for a probable upcoming application. These considerations are in good accordance to those raised by Aris *et al.* who pointed out, that chemicals used in the methods have the ability to react with a broad range of contaminants and might remove even stable contaminants. Whereas less sought after properties of the used materials are rather high costs, the possible formation of precipitates and possible risk to the environment arising from the used materials in the treatment process.^10^ These arguments apply also for the chemical treatments discussed here. For the oxidizing treatment methods, it can be assumed that a broad spectrum of contaminants would react with the used materials, even if seemingly stable molecules are among these contaminants. At the same time, the production of costly catalysts may require an additional investment. Unwanted precipitates can also be expected especially if hardly soluble byproducts are produced. The removal of these precipitates could be carried out mechanically, while soluble byproducts could pose a threat to the environment, independent of the material used for treatment or the targeted contaminant.

### Biological treatment

In total 38 different fungal strains regarding the EE2 removal capability were tested by Różalska *et al.* Pretreated cultures were supplemented with EE2 at a concentration of 10 mg mL^−1^ to determine the reduction efficiency. Also, mineral media were supplemented with various amounts of NaCl to investigate on the effect of NaCl on EE2 removal by *Aspergillus versicolor* IM 2161 and *Aspergillus fumigatus* IM 6510. After 72 h, eighteen of the 38 fungal strains showed a removal efficiency of over 50%. Three strains isolated from soils needed 24 h as a latent period but reached close to 100% removal after 72 h. Other strains achieved almost 100% removal already after 24 h. The presence of sodium chloride had different effects: *A. versicolor* strains showed that both, 0.8% and 1.4% NaCl didn't affect the EE2 removal significantly. 2.8% NaCl caused an initial inhibition, leading to only 17.7% removal after 24 h, but the fungi “caught up” after 48 h. *A. fumigatus* showed an inhibition already at the lowest NaCl concentrations, but the effect was statistically significant for 1.4% and 2.8% NaCl. At the concentration of 2.8% NaCl the removal efficiency accounted to a mere 10% for this strain after 72 h.^43^ Hofmann and Schlosser conducted a study to test the removal of various substances, one of them EE2, using a fungus, namely *Phoma* sp. strain UHH 5-1-03. Pretreated cultures were used to evaluate the removal efficiency in solutions containing 74.1 mg L^−1^ EE2. In addition, supernatants obtained from *Phoma* sp. cultures, which contained laccase as active ingredient, were used. In culture the EE2 content was reduced by 95% after 4 h of incubation while approximately complete removal was achieved after 24 h. The sole supernatant displayed a reduction efficiency of 82% after 4 h of incubation, added syringaldehyde did not affect the EE2 removal. Additional characterization experiments were conducted and showed that during the biological treatment EE2 dimers were produced as degradation products.^44^ Cupuaçu residue, a byproduct from cacao production using *Theobroma grandiflorum*, was tested on its capability regarding EE2 removal *via* induction of laccase release by the fungus *Pycnoporus sanguineus* (ATCC 4518). Experiments were conducted for 24 h in solutions containing 5 mg L^−1^ EE2 and laccase. The highest removal efficiency was measured after 4 h and reached 86.18%. After that, EE2 levels were below the LOD of 0.39 μg mL^−1^, resulting in a maximal removal efficiency >86.18%. Analysis of the degradation products revealed that EE2 dimers and most probably a hydroxylated product was formed in the removal process.^45^ Electrochemically modified dissolved organic matter (DOM) was investigated on by He *et al.* regarding the efficiency of EE2 removal. The researchers modified DOM, separated the fractions based on the molecular weight and tested their respective efficiency on EE2 removal in solutions containing 0.5 mg L^−1^ EE2. In addition, the impact of irradiation, as well as the effect of the addition of a quinone-reducing bacterium *Shewanella oneidensis* was investigated. After 132 h, the highest removal efficiency of 41.6% was reported using 2.9 × 10^9^ CFU mL^−1^*S. oneidensis* MR-1, 5.0 mgC L^−1^ fulvic acids with a molecular weight of below 3 kDa and 1 mmol L^−1^ sodium anthraquinone-2-sulfonate. The authors also stated, that additional photodegradation increased removal rates for EE2 and that the products of the EE2 removal reaction were less toxic than EE2 itself.^46^ An additional study on the EE2 removal using DOM and long-term electro-domesticated microorganisms was conducted by He *et al.*^47^ The highest EE2 removal efficiency was reported with 98.4% after 90 min at an initial concentration of 0.5 mg L^−1^ EE2, using 5.0 mgC L^−1^ fulvic acids (<3 kDa) and 3% (v/v) microorganisms, which were obtained from anaerobic activated sludge at a Chinese purification plant. Further degradation experiments showed that the removal of EE2 was increased after electrical stimulation. A characterization of the degradation products was also performed and showed that EE2 was partially transformed into hydroxylated products as well as estrone and estradiol.^47^ Another study used the bacterium *Hyphomicrobium* sp. GHH and the grass *Lolium perenne* for EE2 removal from soils. Experiments were conducted after spiking soil with EE2 to reach an initial concentration of 23.5 mg kg^−1^. The highest removal efficiency (98.7%) was reported after 42 days using both, *L. perenne* and *Hyphomicrobium* sp. GHH, simultaneously. EE2 was removed from the soil, but was stored in the roots of *L. perenne*.^48^ He *et al.*^49^ conducted a study on the remediation of soil which had been co-contaminated with EE2 and Cd, using ryegrass (which was not further specified in the publication) and *Hyphomicrobium* sp. GHH bacteria. Soil, which has been spiked with 25 mg kg^−1^ EE2 was used and treated with ryegrass, bacteria or both combined for 28 days. Removal rates in combined treatments reached up to 90% when Cd was absent in soil, while removal decreased with increasing Cd concentration. Additional analyses showed that EE2 was mainly stored in the root of the ryegrass well comparable to the results of the aforementioned study.^48,49^

In the biological treatment group, even though only one study was able to report a removal efficiency of approximately 100%,^44^ four other studies reported removal efficiencies of 90% and above^43,46,47,48^ with a shortest treatment period of 90 min. The treatment duration of biological treatment methods was most commonly in the range of hours up to days. In earlier studies mainly ammonia reducing bacteria were used for EE2 degradation,^10^ but more recent studies demonstrate the successful use of fungi or plants for treatment. Fungi cultivation is relatively simple, similarly to bacteria, in contrast to the cultivation of higher plants like *L. perenne* with several more refined requirements. Environmental risks, in particular the production of toxic sludge during EE2 removal, have been a common point of concern in older as well as the recent studies presented. For the studies discussed here, the formation of EE2 dimers^44,45^ or hydroxylated products^47^ should give reason for precaution in further developing these methods. In contrast to Aris *et al.* who reported some studies in which EE2 was often not degradable using biological treatment, from 2014 onwards no study was published that reported that EE2 was not degradable *via* biological treatment. The question that comes up in this case is, if either all researching groups were extremely successful in this field or if studies which were unsuccessful regarding the degradation of EE2 were not published.

### Adsorption and ion exchange

Wang *et al.* assessed the potential of magnetic ion exchange resin (MIEX) to reduce EE2 concentrations. The adsorption experiment was performed for 1 h and after additional 15 min of settling, the EE2 concentration in the supernatant was determined. 75.3% removal efficiency was observed for the lowest initial concentration of 20 μg L^−1^ EE2 at a dosage of 10 mL L^−1^ MIEX. Higher dosages of MIEX did not enhance the removal efficiency. Regarding the extraction mechanism, the authors postulated that “main removal mechanism of EE2 by MIEX was ion exchange instead of reversible micro-pore adsorption”.^50^ They further explained that EE2 molecules diffusing into the resin would encounter an alkaline internal micro-environment, are ionized and form negatively charged molecules which are then removed from water *via* ion exchange.^50^ De Castro *et al.* conducted experiments on the removal of EE2 and other substances using inexpensive materials like sand, vermiculite, non-activated charcoal and granular activated carbon while the latter three all were mixed with sand in polishing units. The effluents of a WWTP were used directly for experiments, which were running without interruption for 2 months with a flow rate of 0.33 m^3^ per day. After 15 days a biofilm had formed, which supposedly was important for the removal efficiency. Samples were taken on ten different days within each experimental run, which lasted 30 days. The initial mean concentration for EE2 was approximately 10 ng L^−1^ and a removal efficiency of over 99% was achieved for every experimental setup.^51^ This study connects to earlier investigations by Snyder *et al.*, who had conducted experiments on the removal efficiency of membranes and activated carbon for pharmaceuticals and endocrine disruptors. EE2 was one of the investigated substances. They reported a removal efficiency of approximately 95% for powdered activated carbon after 4 h at initial concentration of 100 ng L^−1^ EE2.^52^ The removal efficiency for granular activated carbon was over 99% after 30 d at an initial concentration of 10 μg L^−1^. These results are well comparable to the results achieved by de Castro *et al.*^51^ The two teams used different experimental setups and were able to obtain satisfying results with removals of close to 100%.

Tang *et al.* conducted a study focusing on gamma- and beta-cyclodextrin polymers regarding the removal of estradiol, bisphenol A and EE2. The removal of EE2 was tested with an initial concentration of 11.9 μg L^−1^ and the researchers reported a saturation after 10 min for 0.4 mg L^−1^ beta-cyclodextrin polymers and after 5 min for 0.4 mg L^−1^ gamma-cyclodextrin polymers. Approximately 100% removal efficiency were achieved for both polymers after five consecutive regeneration cycles.^53^ Another study which focused on the adsorption of EE2 was conducted by de Oliveira *et al.* using soil as adsorbent. 250 mg of soil were tested in batch experiments mixing soil with 30 mL of a 2 mg L^−1^ EE2 solution for 1440 min. The authors reported an adsorption peak after 45 min corresponding to 27% removal efficiency, which was followed by a decrease of the removal rate, most probably due to consecutive desorption. An equilibrium between adsorption and desorption was reported after 720 min, which equaled approximately 40% of EE2 removal.^54^

Two of the four studies which are in the “adsorption and ion exchange” group showed a removal efficiency of approximately 100%, one achieved this efficiency already after 5 min (ref. 53) and the other after 30 days.^51^ The two other studies showed comparatively lower removal efficiencies of 75.3% and *ca.* 40%, respectively and the duration to achieve those was in between the two previously stated studies. The materials used for the presented studies differ greatly in origin and costs: while soil and sand can be collected easily from the environment and are prepared easily for use, high tech products like MIEX resin and gamma-cyclodextrin polymers cause high costs in purchase and pre-experimental preparation. The works in this group did not degrade EE2 itself but adsorbed or adhered the substance onto a material. In this case the disposal of the material or in case of stripping of EE2 off the material, the stripping matrix should be treated additionally in a way which secures that EE2 is not re-emitted into the environment. Nonetheless, adsorption of EE2 onto material can be classified as a cheaper remediation method if the used material is not expensive, therefore countries which invest less into protection of the environment could implement these methods to prevent the emission of EE2. In contrast to the other treatment groups, adsorption does not necessarily alter the molecular structure of EE2 and therefore it might retain its estrogenicity. After the stripping of EE2 off the adsorbent might be necessary to use another destructive method, *e.g.* the ones from the aforementioned “chemical” or “biological treatment group” to make sure that EE2 is not reemitted into the environment.

As already pointed out by Aris *et al.*^10^ and references therein, advantages of adsorption and ion exchange include high efficiencies and a simultaneous elimination of various substances from aquatic matrices. Disadvantages clearly include the generation of toxic waste, a decrease in removal efficiency over time and non-selective removal (which also might be seen as advantage, depending on the aim of the process) or high costs.

## Conclusions

### EE2 levels in the environment

It appears, that major restrictions for the evaluation of risks associated with EE2 in the environment, in particular in water, are analytical limits. In many cases LODs or LOQs, respectively, have been determined well above the PNEC, which hiders a firm evaluation of risks. Only three of fourteen recent studies reported analytical limits below the PNEC.^10,17,23^ Nonetheless, in studies where EE2 could be identified or quantified, even if the analytical limits were above the PNEC, a strong need for action to reduce environmental concentration of EE2 was shown, if risks for biota should be avoided. For the future, it is recommended to conduct analyses with standardized methods, which reach at least a LOD below 0.035 ng L^−1^. Additionally, degradation products that might possess estrogenic activity need to be identified and included in analyses as well as in risk assessment.

The very different reference matrices *e.g.* dry weight, wet weight or lipid content, presented in the discussed papers does not make it easy to compare data and of course might be a source of insecurity in interpreting them. For better comparison of data, a general agreement or clear guideline for reporting results would be necessary in future.

### Methods for EE2 removal in waste waters

Methods to reduce levels of EE2 in the environment are of different origin and can be grouped in general into chemical treatment, biological treatment, adsorption and ion exchange. Every method has its benefits and disadvantages but shows in general its potential to prevent emissions of EE2 into the environment. Depending on the initial concentration of EE2, the desired treatment time and expected removal efficiency, various methods are available for use and worth being further developed.

Most studies that report removal efficiencies of close to 100% or even “100%” straight, do not state a LOD or LOQ. Which leads us back to the call for standardized analytical methods and the urge to report analytical limits. Comparing all methods presented, it has to be stated, that a feasible method for the treatment of larger quantities of waste water has not been established yet. Most promising in this regard seems the use of unspecific adsorption onto cheap and reusable materials combined with a chemical treatment for a complete destruction of organic components and the consequent regeneration of the adsorbent. Following those suggestions could lead to an easier evaluation of methods and more success in efforts to reduce EE2 in our environment.

## Conflicts of interest

There are no conflicts to declare.

## Supplementary Material
